# Parametric MRI Detects Aristolochic Acid Induced Acute Kidney Injury

**DOI:** 10.3390/tomography8060243

**Published:** 2022-12-10

**Authors:** Yingjie Mei, Guixiang Yang, Yihao Guo, Kaixuan Zhao, Shuyu Wu, Zhongbiao Xu, Shan Zhou, Chenggong Yan, Erdmann Seeliger, Thoralf Niendorf, Yikai Xu, Yanqiu Feng

**Affiliations:** 1 School of Biomedical Engineering, Southern Medical University, Guangzhou 510515, China; 2 Department of Medical Imaging Center, Nanfang Hospital, Southern Medical University, Guangzhou 510515, China; 3 Department of Radiology, Hainan General Hospital (Hainan Affiliated Hospital of Hainan Medical University), Haikou 570311, China; 4 Radiotherapy Center, Affiliated Cancer Hospital & Institute of Guangzhou Medical University, Guangzhou 510095, China; 5 Radiotherapy Center, Guangdong General Hospital, Guangzhou 510080, China; 6 State Key Laboratory of Organ Failure Research, National Clinical Research Center of Kidney Disease, Division of Nephrology, Nanfang Hospital, Southern Medical University, Guangzhou 510515, China; 7 Institute of Translational Physiology, Charité–Universitätsmedizin Berlin, 10117 Berlin, Germany; 8 Berlin Ultrahigh Field Facility (B.U.F.F.), Max Delbrück Center for Molecular Medicine in the Helmholtz Association, 13125 Berlin, Germany; 9 Guangdong Provincial Key Laboratory of Medical Image Processing & Guangdong Province Engineering Laboratory for Medical Imaging and Diagnostic Technology, Southern Medical University, Guangzhou 510515, China; 10 Guangdong-Hong Kong-Macao Greater Bay Area Center for Brain Science and Brain-Inspired Intelligence & Key Laboratory of Mental Health of the Ministry of Education, Southern Medical University, Guangzhou 510515, China; 11 Department of Radiology, Shunde Hospital, Southern Medical University (The First People’s Hospital of Shunde, Foshan), Foshan 528399, China

**Keywords:** magnetic resonance imaging, T_2_ mapping, diffusion weighted imaging, aristolochic acid, acute kidney injury

## Abstract

Exposure to aristolochic acid (AA) is of increased concern due to carcinogenic and nephrotoxic effects, and incidence of aristolochic acid nephropathy (AAN) is increasing. This study characterizes renal alterations during the acute phase of AAN using parametric magnetic resonance imaging (MRI). An AAN and a control group of male Wistar rats received administration of aristolochic acid I (AAI) and polyethylene glycol (PEG), respectively, for six days. Both groups underwent MRI before and 2, 4 and 6 days after AAI or PEG administration. T_2_ relaxation times and apparent diffusion coefficients (ADCs) were determined for four renal layers. Serum creatinine levels (sCr) and blood urea nitrogen (BUN) were measured. Tubular injury scores (TIS) were evaluated based on histologic findings. Increased T_2_ values were detected since day 2 in the AAN group, but decreased ADCs and increased sCr levels and BUN were not detected until day 4. Significant linear correlations were observed between T_2_ of the cortex and the outer stripe of outer medulla and TIS. Our results demonstrate that parametric MRI facilitates early detection of renal injury induced by AAI in a rat model. T_2_ mapping may be a valuable tool for assessing kidney injury during the acute phase of AAN.

## 1. Introduction

The exposure to aristolochic acid (AA) has become an increasing concern due to its mutagenic, carcinogenic and nephrotoxic effects [1,2,3]. AAs are a group of compounds found in plants in the family Aristolochiaceae. These plants have long been used for various medicinal purposes, such as arthritis, abscesses, inflammation and chronic pain [2,4]. Millions of people have been affected by aristolochic acid nephropathy (AAN), which is recognized as a toxic interstitial nephropathy caused by ingestion of plants containing AA [5]. Despite measures that regulate AA consumption in many countries and regions, cases of AAN continue to occur especially in areas where traditional medicine is popular [6]. It is estimated that more than 100 million people are at risk of developing AAN worldwide [7]. AAN was demonstrated a biphasic evolution of renal injury, with the first acute phase characterized by signs of proximal tubular cell swelling and necrosis [8,9,10,11,12]. The early diagnosis of acute kidney injury (AKI) induced by AA is of great clinical importance for disease management and effective therapeutic intervention [13,14]. Despite their well-known shortcomings, serum creatinine (sCr), a surrogate marker for glomerular filtration rate (GFR), and sCre-based estimated GFR (eGFR) are still used as the standard point-of-care metrics for the diagnosis of AKI in the vast majority of medical settings, in accordance with current clinical practice guidelines [15,16]. Their diagnostic clinical utility in the case of AAN is, moreover limited, as they are surrogate markers for GFR rather than markers for injury of proximal tubules. Histologic examination remains the mainstay for diagnosis of AAN, which is invasive and susceptible to sampling error [4,13]. Therefore, a reliable non-invasive biomarker that aims for early diagnosis, disease progression monitoring and prognosis of AAN is of urgent clinical need [4].

Magnetic resonance imaging (MRI) plays an important role in kidney disease research, for not only providing excellent anatomical images but also being useful in probing renal pathophysiology using functional renal MRI techniques, such as blood oxygenation level-dependent (BOLD) image contrast, water diffusion weighted imaging (DWI), and magnetic resonance relaxometry, etc. [17,18,19,20]. Quantitative mapping of the relaxation time T_2_ is a well-established technique for (pre)clinical research. T_2_ is sensitive to both the free water content in tissue and the amount of paramagnetic deoxygenated hemoglobin (deoxyHb) per tissue volume (voxel). Therefore, T_2_ mapping allows the assessment of the degree of tissue edema as well as probing renal BOLD [21,22,23,24,25,26,27,28,29]. DWI, which probes the tissue microstructure through the displacement of water molecules, has been shown to provide specific information on renal tissue and is considered to be a biomarker of MR renal functional imaging [30,31,32,33]. 

Previous studies demonstrated that changes of renal T_2_ mirror characteristic pathophysiologic changes in the course of AKI development and its possible progression to chronic kidney disease (CKD). These include tissue hypoperfusion and hypoxia as observed, e.g., in the initial reperfusion phase following renal ischemia and upon administration of an x-ray contrast agent, edema formation and inflammatory infiltration, and finally, fibrosis [32,34,35]. In a study of kidney transplantation, the abrogation of the physiological gradient of T_2_ across the kidney reflected the loss of tubular function and identified kidney grafts with acute rejection [36]. Restricted water diffusion, which manifests itself in a reduction of the apparent diffusion coefficient (*ADC*), has been reported in AKI when the interstitial space was narrowed due to infiltration of inflammatory cells or fibrosis [37,38,39]. 

Recognizing the clinical need for diagnosis, disease management and therapy of AAN, this study examines the applicability of quantitative MRI of T_2_ and *ADC* for the assessment of early renal microstructural alterations and proximal tubular injury induced by AA. 

## 2. Materials and Methods

### 2.1. Experiment Protocols

All animal experiments were performed under a protocol approved by local Animal Ethics Committee (IACUC No.: L2018132). Twenty-eight male Wistar rats with an age of 5 to 6 weeks and an initial weight ranging from 140–160 g were obtained from the Animal Center of our institute. All rats were kept in cages, housed in an animal room with stable environment (i.e., temperature, humidity and 12 h-cycle light) and provided with drinking water and rat chow ad libitum. 

**Figure 1:**
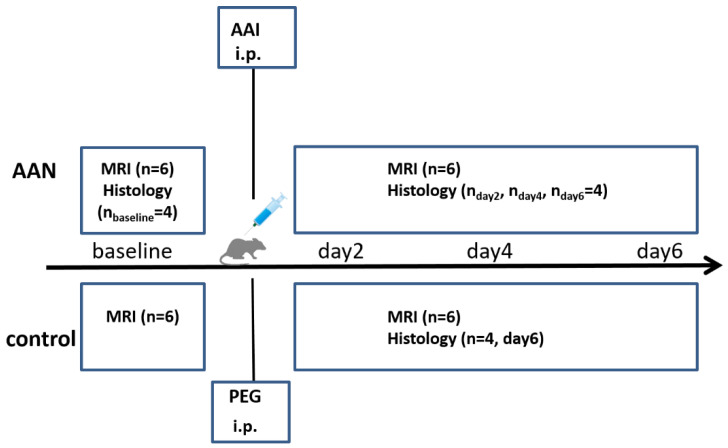
Schematic representation of the experimental protocol performed in rats involving an AAN group and a control group. Kidneys and blood samples were collected after each MRI examination of the AAN group, and after the last MRI examination in the control group. AAN: aristolochic acid nephropathy; AAI: aristolochic acid I; i.p.: intraperitoneal injection; PEG: polyethylene glycol.

Figure 1 shows the schematic representation of experiment protocols performed in rats of the AAN group and the control group. Weight-matched rats were randomly assigned to AAN group (n = 22) and control group (n = 6). A previously described acute AAN model was used to induce AKI [11]. The AAN group received daily intraperitoneal injections of 40 mg/kg AAI ((Sigma-Aldrich, St. Louis, MO, USA) dissolved in polyethylene glycol (PEG) for six days. The control group received daily intraperitoneal injections of PEG the same dosage and schedule as the AAN group.

The rats were fastened 12 h before the MRI examination which was performed (at a set time of the day) for six rats from each group before, at day 2, day 4 and day 6 after AAI or PEG administration. Kidney and blood samples were collected from four rats after each MRI examination for the AAN group and after the last MRI examination for the control group.

### 2.2. Data Acquisition

MRI was conducted on a 7 Tesla animal scanner (Pharma Scan, Bruker Biospin, Ettlingen Germany) using a volume RF coil for transmission and 4-channel rat surface RF coil for signal reception. Rats were placed in supine position to ensure that kidneys were close to the RF coil and to reduce kidney movement caused by respiration. Anesthesia was conducted by means of inhalation of isoflurane, 5% for seduction and 2% for maintenance. Respiration was monitored and kept at a rate of 60 respiration cycles per minute using a ventilator. A circulating water blanket maintained at 38 °C was placed on the belly. High spatial resolution T_2_ weighted 3D Fast Spin Echo (FSE) imaging (TR/TE = 3000/66 ms; RARE factor = 20; FOV = 40 × 55 × 4 mm^3^; matrix = 200 × 275 × 20; voxel size = 0.2 × 0.2 × 0.2 mm^3^; number of averages = 1; scan duration = 13 min) was first performed for anatomical imaging. Respiratory triggering was employed to reduce respiratory motion induced artifacts. 3D T_2_-weighted images were acquired in coronal orientation placed parallel to both kidneys. A 2D Carr–Purcell–Meiboom–Gill (CPMG) multi-echo spin-echo sequence was performed for quantitative T_2_ mapping (TR/ΔTE/first TE = 1000/10/10 ms, number of echoes = 8; FOV = 40 × 55 mm^2^; matrix size = 133 × 183; slice thickness = 1 mm; number of slices = 8; number of averages = 2; scan duration = 6 min). For *ADC* mapping, single shot echo-planar DWI was applied (diffusion weighting b = 0 and 800 s/mm^2^, TR/TE = 3000/24 ms; FOV = 45 × 55 mm^2^; matrix size = 128 × 96; receiver bandwidth = 1562 Hz/pixel, slice thickness = 1 mm; number of averages = 4; acceleration factor = 2).

### 2.3. MR Image Analysis

T_2_ maps were calculated by a pixel-wise fitting using a mono-exponential fitting algorithm based on auto-regression on linear operation (ARLO) of multi-echo data [40] with Matlab 2014 software (MathWorks, Natick, MA, USA). 

The principal component analysis denoising method was used for noise suppression on the original DWI images [41]. *ADC* maps were obtained by pixel-wise fitting of the diffusion sensitized images to Equation (1) using non-linear least squares method:

(1) $$S_{b}=S_{0}\cdot e^{-ADC\cdot b}$$

where *S_b_* and *S*_0_ denote the MR signals with and without diffusion weighting, and *b* is the diffusion weighting strength. Regions of interest (ROIs) were defined for the renal cortex (CO), the outer stripe of outer medulla (OSOM), the inner stripe of outer medulla (ISOM) and inner medulla (IM). The ROIs were manually drawn on the right kidney. Mean values of T_2_ and *ADC* were estimated for each renal layer.

### 2.4. Biochemical Measurements and Histologic Evaluations

Blood samples were collected from the aorta and centrifuged at 3000 r/min for 10 min at 4 °C. The serum was stored at −80 °C for sCr and blood urea nitrogen concentration (BUN) measurements. sCr and BUN levels were measured using an automatic biochemical analyzer (AU480; Beckman Coulter, Pasadena, CA, USA). 

Kidneys were harvested and fixed in formalin after being perfused with saline via the left ventricle while rats were under general anesthesia. Formalin fixed tissues were embedded in paraffin. Longitudinal sections were cut at a slice thickness of 2 µm and stained with hematoxylin and eosin to evaluate glomerular and tubular injury. Semi-quantitative determination of the tubular injury score (TIS) was carried out by methods reported previously [42,43]. Briefly, the tubular injury included the following categories: no injury (0), tubular epithelial cell flattening (1), brush border loss (1), cell membrane bleb formation (1–2), cytoplasmic vacuolization (1), cell necrosis (1–2), tubular lumen obstruction (1–2). Scores for all categories were accumulated for final tubular injury scoring with the maximum score being nine. 

### 2.5. Statistical Analysis

Statistical analyses were carried out using SPSS 22.0 (IBM, New York, NY, USA). Data are presented as mean ± standard error of the mean (SEM). Longitudinal changes in T_2_ and *ADC* after AAI injection were assessed by using one-way analysis of variance (ANOVA) for repeated measurements followed by post hoc multiple comparisons. Differences in T_2_ and *ADC* between the AAN group and the control group were evaluated using unpaired *t* test. Correlations between TIS and MR parameters were determined by calculating the Spearman correlation coefficient. A *p* value lower than 0.05 was considered to be significant.

## 3. Results

**Figure 2:**
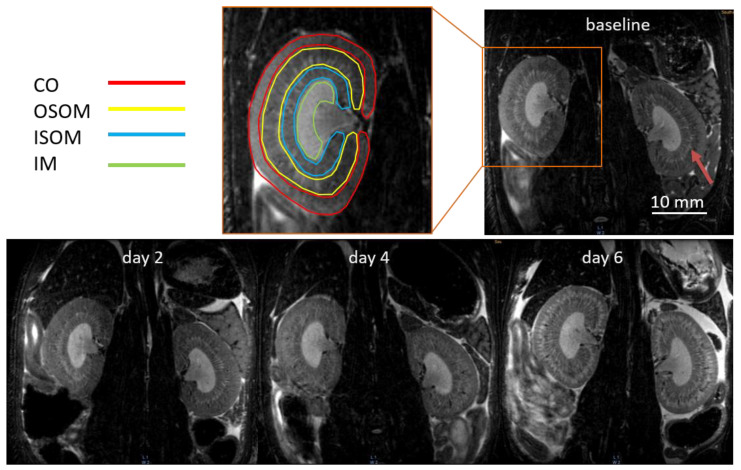
Examples of isotropic T2-weighted images of AAI-treated kidneys. At baseline, four kidney layers are outlined with contours of different colors in the zoomed image. CO, cortex; OSOM, outer stripe of outer medulla; ISOM, inner stripe of outer medulla; IM, inner medulla.

High spatial resolution 3D T_2_-weighted MRI revealed structural changes of AAI-treated kidneys at different time points (Figure 2). Four renal layers can be distinguished with good tissue contrast at baseline. The contours are outlined with different colors in the zoomed image. The isotropic resolution of 0.2 mm even facilitated the visualization of the collecting ducts in the outer medulla (arrow). After the administration of AAI, the corticomedullary T_2_-weighted contrast differentiation (CMD) diminished at day 2 and day 4. The collecting ducts in the medulla could not be observed either at day 4. At day 6, the OSOM show marked hyperintensity compared to CO and ISOM. No distinct changes of IM were observed from T_2_-weighted images. 

**Figure 3:**
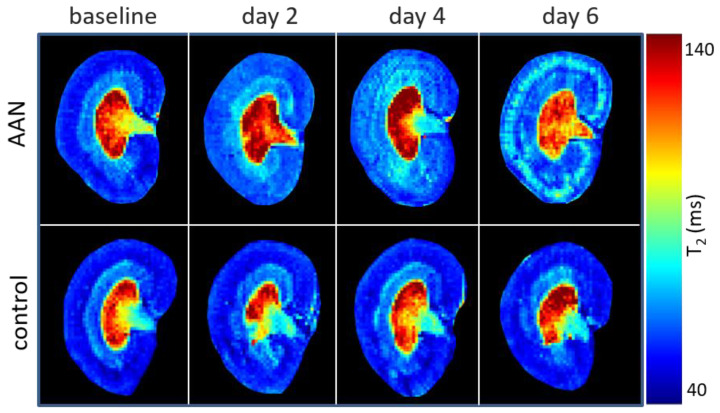
Representative renal T2 maps observed for the AAN group (**upper row**) and for the control group (**bottom row**).

**Figure 4:**
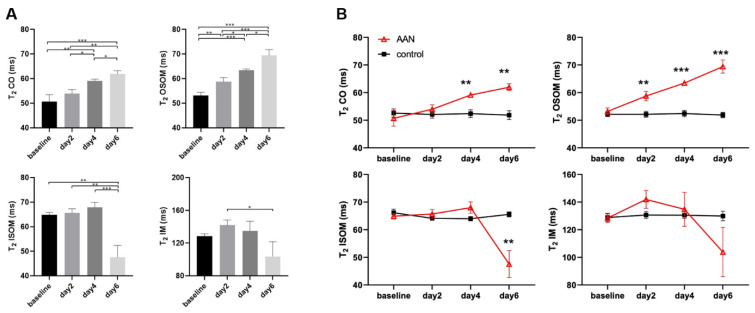
Statistical analysis of T2 relaxation times for the AAN group (**A**) and the control group (**B**). *** *p* < 0.001, ** *p* < 0.01, * *p* < 0.05. CO, cortex; OSOM, outer stripe of outer medulla; ISOM, inner stripe of outer medulla; IM, inner medulla.

Representative T_2_ maps of the AAN (upper row) and the control group (lower row) are illustrated in Figure 3. For kidneys without AAI, T_2_ showed an increased gradient from CO to IM. For the AAN group, T_2_ of CO and OSOM were increased to day 6. The outcome of the statistical analysis of T_2_ of the AAN group is shown in Figure 4A. The T_2_ increase of OSOM was immediately detected at day 2 (baseline vs. day 2 *p* = 0.005), while that of CO was not detected until day 4 (baseline vs. day 4 *p* < 0.006). T_2_ of both layers were increased remarkably at day 6 (CO: baseline vs. day 6 *p* < 0.001; OSOM: baseline vs. day 6 *p* < 0.001). OSOM present the most pronounced T_2_ change: T_2,day2_ = 53.2 ms to T_2,day6_ = 69.4 ms. T_2_ of ISOM and IM increased until day 4 followed by a sudden decline observed at day 6, showing T_2_ relaxation times being smaller than at baseline. 

The comparison between T_2_ derived from the AAN and the control group is shown in Figure 4B. For the control group, T_2_ remained stable from baseline to day 6 for all four renal layers. T_2_ of CO and OSOM was significantly higher in AAN than in the control group at day 4 and day 6 (CO: day 4 *p* = 0.002 and day 6 *p* < 0.001; OSOM: day 4 *p* = 0.009, day 6 *p* < 0.001 and *p* < 0.001). ISOM showed a T_2_ reduction (*p* = 0.013) only at day 6 if benchmarked against the control group. For IM, no significant T_2_ difference was detected between the AAN group and the control group. The T_2_ relaxation times of the AAN and the control group are summarized in Table 1.

**Figure 5:**
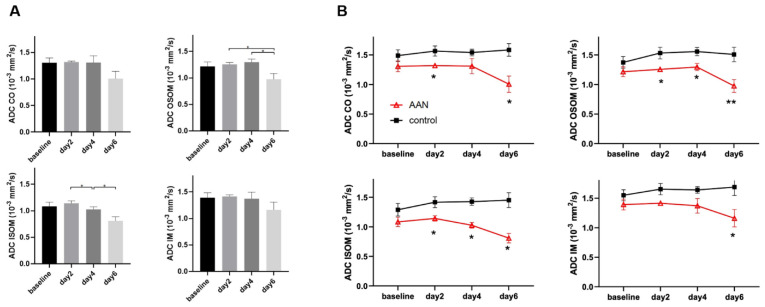
Statistical analysis of apparent water diffusion coefficients (ADCs) for the AAN group (**A**) and the control group (**B**). ** *p* < 0.01, * *p* < 0.05. CO, cortex; OSOM, outer stripe of outer medulla; ISOM, inner stripe of outer medulla; IM, inner medulla.

The ADCs obtained for the AAN and the control group are summarized in Table 2. The results derived from the statistical analysis of the ADCs of the AAN group are shown in Figure 5A. *ADC* of OSOM was significantly reduced at day 6, while *ADC* of ISOM was significantly reduced from day 4. The time course of CO and IM showed no significant changes. The *ADC* comparison between the AAN and the control group is illustrated in Figure 5B. The control group showed no *ADC* change from baseline through day 6. The AAN group yielded ADCs lower than those of the control group, particularly in OSOM and ISOM. 

**Figure 6:**
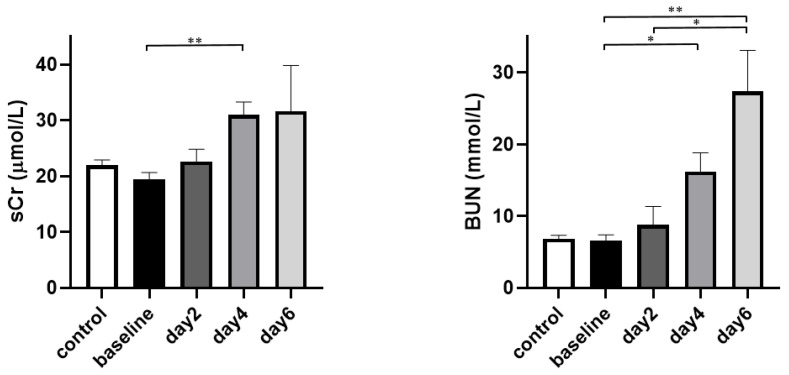
Serum creatinine levels (sCr) (**left**) and blood urea nigrogen (BUN) (**right**) of the control group and the AAI group. ** *p* < 0.01, * *p* < 0.05.

The results of sCr levels and BUN are shown in Figure 6. No difference in sCr was observed between the control group and the baseline of the AAN group. For the AAN group, an increased sCr level was only detected at day 4 (control vs. day 4 *p* = 0.009, AAN baseline vs. day 4 *p* = 0.005). The increase of BUN was not detected until day 4 (control vs. day 4 *p* = 0.012, control vs. day 6 *p* = 0.008, AAN baseline vs. day 4 *p* = 0.012, AAN baseline vs. day 6 *p* = 0.008). 

**Figure 7:**
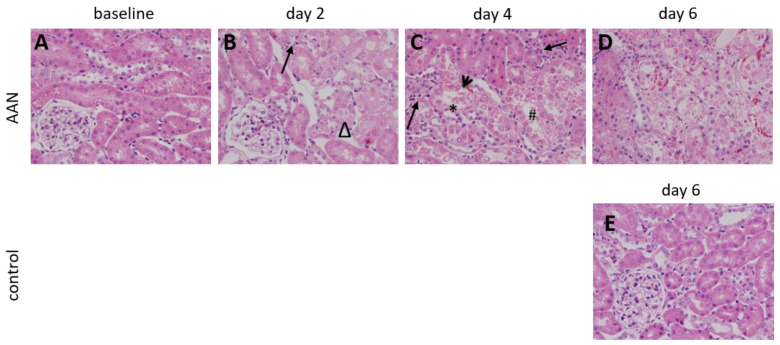
Photomicrographs (magnification, ×400) of hematoxylin–eosin staining reveal renal tissue injuries for the AAN group at baseline (**A**), day 2 (**B**), day 4 (**C**) and day 6 (**D**). At day 2, renal alterations were characterized by vacuolization and swelling of the proximal tubular epithelial cells (Δ). At day 4, tubular necrosis (arrow head), loss of brush borders (*) and fragments of tubular epithelial cells (#) were observed. The above tubular damages became massive and more serious at day 6. No signs of regeneration were observed. Inflammatory cell infiltration was detected (long arrow). No histological changes were found in the control group (**E**).

Renal tissue injuries of the AAN and the control group were investigated with hematoxylineosin staining (Figure 7). At baseline, examination of all kidney samples showed no significant abnormalities before treatment (Figure 7A). For the AAN group, the histologic lesions were mainly presented in proximal tubules. At day 2, the changes of the proximal tubular epithelial cells were characterized by vacuolization and swelling (Figure 7B, Δ). Inflammatory cell infiltration was detected (long arrow). At day 4, tubular necrosis (arrow head), loss of brush borders (*) and fragments of tubular epithelial cells (#) were observed (Figure 7C). The above tubular damages became diffusive at day 6 (Figure 7D). No signs of regeneration or interstitial fibrosis were observed. No histologic changes were found in the control group at day 6 (Figure 7E). The glomeruli remained intact in both groups. The AAN group revealed an averaged TIS_baseline_ = 0, TIS_day2_ = 3, TIS_day4_ = 6 and TIS_day6_ = 8. For the control group TIS = 0 was found.

**Figure 8:**
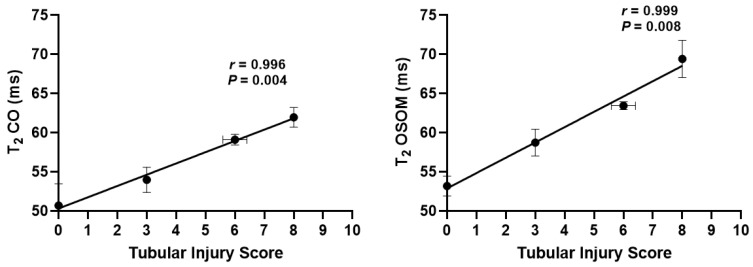
Correlations between tubular injury scores (TIS_baseline_ = 0, TIS_day2_ = 3, TIS_day4_ = 6 and TIS_day6_ = 8) and MRI parameters. CO, cortex; OSOM, outer stripe of outer medulla.

The correlations between TIS and MR parameters are illustrated in Figure 8. Significant linear correlations were observed between TIS and T_2_ of CO (*r* = 0.996, *p* = 0.004) and OSOM (*r* = 0.999, *p* = 0.008). No correlation was observed between TIS and *ADC*.

## 4. Discussion

This study examined the feasibility of parametric MRI for the assessment of renal injury in an AAI-treated rat model using the quantitative MRI metrics T_2_ and *ADC*. T_2_-weighted MRI was able to separate four renal layers from each other with good tissue contrast and showed microstructural changes of different renal layers at each time point. Severe renal injury was detected with *ADC* mapping. T_2_ of OSOM and CO presents changes earlier than the standard clinical metrics (sCr and BUN) for diagnosing AKI, and was significantly correlated with TIS. 

T_2_ mirrors free water content in tissue and the amount of deoxyHb per tissue volume. Thus, an increase of T_2_ may result from increased free water, e.g., due to edema formation, a decrease in deoxyHb, or a combination hereof. Lowered blood oxygenation (i.e., decreased O_2_ saturation of Hb) and a decrease in the blood volume fraction result in decreased deoxyHb per tissue volume. The latter can be induced by active vasoconstriction and by passive compression of intrarenal vessels. It is most probable that the observed changes in T_2_ represent the sum of all these effects. In the face of the relatively rigid renal capsule, edema formation and influx of immune cells will result in an ‘intrarenal compartment syndrome’: as intrarenal pressure increases, intrarenal blood vessels become compressed [29,44,45,46]. Studies on AAN revealed that the intoxication results in intrarenal vasoconstriction due to acute deficiency of vasodilatory nitric oxide (NO) [47]. Besides its effect on T_2_, passive compression and active vasoconstriction diminish O_2_ supply, thereby resulting in tissue hypoxia. Renal tissue hypoperfusion and hypoxia are a pivotal early element in the pathophysiology of AKI of various origins as well as for the promotion from AKI to CKD [48,49,50,51,52,53].

In order to improve the delineation of the rat kidney, an organ with a size about 10 × 4 × 3 mm, 3D fast spin echo T_2_-weighted imaging with 0.2 mm isotropic spatial resolution was employed. At day 2 and day 4, the obscurity of the distinguishable CMD can be attributed to changes in T_2_ that were more pronounced in the OSOM versus the CO. This could rely on a pronounced edema formation and/or pronounced decrease in deoxyHb in OSOM. It must be noted that the outer medulla (OM) is the area at risk in AKI, due to its sparse O_2_ delivery compared to the high O_2_ demands for active solute transports in the thick ascending limb of the loop of Henle and in the pars recta of the proximal tubule [48,49,53]. Therefore, the vicious cycle of hypoxia, cell damage, edema formation, and vessel compression in OM is pronounced versus the other layers. The marked hyperintensity obtained for OSOM at day 6 probably mirrors the further progression of these pathophysiologic events. In the current study, T_2_-weighted imaging with high spatial resolution and sufficient SNR has shown potential for the assessment of structural and blood oxygenation changes induced by AA.

The contrast changes presented on T_2_-weighted images of different renal layers could be further clarified and confirmed by the changes derived from T_2_ mapping. Among the four renal layers, T_2_ time course of OSOM showed the earliest change, followed by the T_2_ time course of CO. T_2_ of OSOM and CO were increased monotonically afterwards. In particular, T_2_ of OSOM showed the most pronounced changes. It was reported in previous studies that the proximal tubule was the main target of AA, while the glomerulus was rarely found injured at early stages of AAN [9,10,48]. Our histologic analysis revealed that injury to the proximal tubules was mainly observed for OSOM, with some spatial extension to CO. This finding suggests that the rise of T_2_ in OSOM and CO is in part attributable to proximal tubular epithelial cell swelling and inflammatory cells observed in histologic analysis. In addition, T_2_ obtained for OSOM and CO revealed significant correlations with TIS. This indicates that T_2_ mapping might be a useful technique for the assessment of AA-induced renal injury. 

Similar to previous studies, in which T_2_ found for OSOM (or OM) showed the most pronounced changes after ischemia/reperfusion injury (I/R) [32,34,35], our study demonstrated the pronounced T_2_ changes upon AA intoxication for OSOM. While part of the underlying mechanisms differ, e.g., the primary toxic effect in AAN, our observations confirm that OM is the most vulnerable layer of the kidney in various forms of AKI. Our T_2_ time courses of AAI treated kidneys showed no signs of decline within the observation period. This finding is in line with clinical observations: in many patients, AAN turns out to be a rapidly progressing disease [54]. 

The ADCs obtained for the AAN group were significantly lower than those of the control group, especially for OSOM and ISOM. Although the ADCs of the AAN group showed a decrease over time, significant differences between the time points were barely detected. *ADC* reduction obtained for OSOM at day 6 and ISOM at day 4 constitute an exception. Significant *ADC* reduction and T_2_ prolongation suggest severe renal tissue damage at these time points. In previous studies, *ADC* reduction after AKI was reported to be associated with cell swelling and infiltration of inflammatory cells into the interstitial space [32,36,55]. Our results demonstrated *ADC* changes of OSOM only at day 6 after the intoxication of AAI. This indicates a decrease in the interstitial water fraction only at this stage of AAI intoxication. No correlation between *ADC* and TIS was observed. We hypothesize that several factors may have contributed to the insensitivity of *ADC* during the first 4 days. First, cell density may need to increase to a certain level to be detected by *ADC* changes. Second, besides apparent water diffusion, *ADC* also reflects blood flow in the microcirculation and tubular fluid flow in the kidney [38,39]. Hence, more sophisticated DWI models that deal with water diffusion, microcirculation and tubular flow separately such as IVIM or less constrained, data-driven non-negative least squares (NNLS) continuum approaches for renal DWI analysis should be employed in future studies [56,57,58,59].

In accordance with current clinical practice guidelines, in the vast majority of medical settings, the diagnosis of AKI is still based on the surrogate parameters for GFR, sCr and BUN. The elevation of these metrics was not detected until day 4 (sCr at day 4 and BUN at day 6) when tubular necrosis was shown. T_2_ changes occurred earlier and showed significant elevation at day 2 when vacuolization and swelling of proximal tubular epithelia cells were observed. This observation is consistent with previous reports concluding that the assessment of kidney injuries with blood tests is feasible when kidney function was impaired severely [60,61,62,63]. Unlike sCr and BUN results, T_2_ obtained for OSOM differed significantly among all time points. The well-known shortcomings of sCr and BUN for the detection of AKI of all etiologies include their low sensitivity, their exceptionally sluggish response related to their large volume of distribution, and the fact that they do not mirror early pivotal events in the pathophysiology of AKI [64,65]. Thus, T_2_ mapping is conceptually appealing for monitoring progression of AA-induced disease in its initial phase. Our study demonstrated another advantage of T_2_ mapping over sCr and BUN because T_2_ mapping enables layer-specific and even voxel-based evaluation of kidney alterations. 

T_2_ showed a significant positive correlation with TIS, of which T_2_ of OSOM revealed the highest correlation coefficient. This finding deems T_2_ a suitable imaging marker candidate for the assessment of tubular injury during the acute phase of AAN.

A recognized limitation of our study is that the MRI examination covered only day 2 to day 6 after AAI administration. Recognizing the T_2_ alterations in OSOM observed at day 2 already, we advise to use an earlier time point for further validation of the efficacy of renal T_2_ mapping for the earliest possible detection of renal injury of AAN. Likewise, time points beyond day 6 should be studied in order to elucidate the further pathophysiologic events that lead to fibrosis and, ultimately, to CKD. It is an additional recognized limitation of our study that the sample size of the biochemical and histologic evaluations is relatively small.

In summary, parametric MRI facilitates assessment of microstructural and functional renal alterations induced by AA. MRI-derived parameters, in particular T_2_, enables detection of AAN earlier than the blood-based metrics used in today’s standard AKI diagnostics. Our results, furthermore demonstrate that high spatial resolution morphological T_2_-weighted MRI supports visualization of early structural changes. T_2_ mapping is more sensitive than DWI for the monitoring of early alterations and progression of kidney injury following AAI intoxication. This finding underlines the potential of T_2_ mapping as a non-invasive imaging marker for the in vivo assessment of renal alterations during the early phase of AAN. 

## Figures and Tables

**Table 1:** Summary of T_2_ relaxation times obtained for the AAN group and for the control group.

T_2_ (ms)	Group	Baseline	Day 2	Day 4	Day 6
CO	AAN	50.69 ± 2.79	53.98 ± 1.59	59.11 ± 0.68 **	61.96 ± 1.25 ***
	control	52.57 ± 1.51	52.07 ± 1.33	52.39 ± 1.39	51.85 ± 1.59
OSOM	AAN	53.19 ± 1.26	58.74 ± 1.71 *	63.44 ± 0.49 ***	69.43 ± 2.37 ***
	control	52.16 ± 0.85	52.15 ± 1.07	52.42 ± 1.13	51.88 ± 0.98
ISOM	AAN	64.87 ± 0.97	65.66 ± 1.63	67.99 ± 2.04	47.55 ± 4.84 *
	control	66.14 ± 1.31	64.10 ± 0.46	63.99 ± 0.79	65.53 ± 0.90
IM	AAN	128.41 ± 3.01	141.91 ± 6.44	134.73 ± 12.21	103.79 ± 17.89
	control	128.85 ± 2.96	130.65 ± 2.52	130.52 ± 2.40	129.89 ± 3.47

*** *p* < 0.001, ** *p* < 0.01, * *p* < 0.05, compared with control rats. CO, cortex; OSOM, outer stripe of outer medulla; ISOM, inner stripe of outer medulla; IM, inner medulla.

**Table 2:** Summary of apparent water diffusion coefficients (*ADC*) obtained for the AAN group and for the control group.

*ADC* (10^−3^ mm^2^/s)	Group	Baseline	Day 2	Day 4	Day 6
CO	AAN	1.307 ± 0.09	1.319 ± 0.018 *	1.311 ± 0.127	1.006 ± 0.139 *
	control	1.489 ± 0.099	1.566 ± 0.086	1.541 ± 0.056	1.585 ± 0.108
OSOM	AAN	1.215 ± 0.083	1.256 ± 0.035 *	1.295 ± 0.061 *	0.975 ± 0.109 **
	control	1.374 ± 0.098	1.532 ± 0.098	1.557 ± 0.069	1.508 ± 0.121
ISOM	AAN	1.083 ± 0.079	1.142 ± 0.045 *	1.027 ± 0.048 *	0.809 ± 0.08 *
	control	1.289 ± 0.106	1.417 ± 0.092	1.426 ± 0.062	1.451 ± 0.124
IM	AAN	1.393 ± 0.091	1.416 ± 0.03	1.372 ± 0.124	1.161 ± 0.146 *
	control	1.55 ± 0.094	1.654 ± 0.095	1.64 ± 0.055	1.688 ± 0.141

** *p* < 0.01, * *p* < 0.05, compared with control rats. CO, cortex; OSOM, outer stripe of outer medulla; ISOM, inner stripe of outer medulla; IM, inner medulla.
